# Gut microbiota-derived formate exacerbates pulmonary metastasis in cancer

**DOI:** 10.7150/thno.108873

**Published:** 2025-07-02

**Authors:** Ray Putra Prajnamitra, Chaw Yee Beh, Wen-Hung Tang, Shu-Chian Ruan, Yuan-Yuan Cheng, Pei-Teng Kuo, Yu-Wen Tien, Patrick Ching-Ho Hsieh

**Affiliations:** 1 Institute of Biomedical Sciences, Academia Sinica, Taipei 115, Taiwan.; 2 Department of Surgery, National Taiwan University Hospital and National Taiwan University College of Medicine, Taipei 100, Taiwan.; 3 Institute of Medical Genomics and Proteomics and Institute of Clinical Medicine, National Taiwan University College of Medicine, Taipei 100, Taiwan.

**Keywords:** Melanoma, lung metastasis, formate, metabolomics, microbiota

## Abstract

**Rationale:** The gut microbiota and its metabolites significantly influence cancer development and metastasis. Among these, formate, the simplest short-chain fatty acid (SCFA), remains underexplored in the context of metastasis. This study investigates the role of microbiota-derived formate in exacerbating pulmonary metastasis in melanoma and pancreatic ductal adenocarcinoma (PDAC), aiming to elucidate its mechanistic contributions to cancer progression.

**Methods:** Using antibiotics-induced dysbiosis in mice, we quantified plasma formate levels via nuclear magnetic resonance (NMR) metabolomics and identified gut bacterial contributors through 16S rRNA sequencing. Formate's effects on melanoma and PDAC lung metastases were evaluated through *in vivo* supplementation experiments. Cellular assays, metabolomics, and gene expression analyses further elucidated its mechanistic impact.

**Results:** Dysbiosis significantly increased circulating formate levels, with *Enterobacterales* identified as key contributors. Formate supplementation enhanced melanoma and PDAC lung metastases by promoting cancer cell proliferation, migration, and nucleotide synthesis. Mechanistic studies revealed that formate upregulated one-carbon metabolism, critical for tumor aggressiveness, and increased the production of metabolites like glutathione, facilitating oxidative stress resistance.

**Conclusion:** Microbiota-derived formate plays a critical role in enhancing pulmonary metastasis by modulating cancer cell metabolism. These findings highlight the therapeutic potential of targeting formate production or its associated metabolic pathways to mitigate cancer spread. Additionally, microbiome modulation emerges as a promising complementary approach to improving cancer treatment outcomes.

## Introduction

In recent years, the role of the gut microbiota and its metabolites has emerged as a fascinating area of research in the field of cancer biology. Evidence suggests that the gut microbiota and its metabolites have a significant impact on the development, progression, and treatment of cancer 1-3. Numerous studies have revealed intriguing associations between alterations in the gut microbiota composition, known as dysbiosis, and the occurrence of various types of cancer, such as colorectal 4-6, liver 7, 8, skin 9, 10, and breast cancers 11-13. These findings have sparked intense interest in understanding the mechanisms underlying the interactions between the gut microbiota and cancer.

The gut microbiota produces a diverse array of metabolites through the fermentation of dietary components, such as carbohydrates and fibres 14, 15. These metabolites, including short-chain fatty acids (SCFAs) and secondary bile acids have been found to have both beneficial and detrimental effects on cancer development. For instance, SCFAs, such as acetate 16, 17 is pro-tumorigenic, whilst propionate 18 and butyrate 19 are anti-tumorigenic. Furthermore, microbial metabolites are also linked to cancer metastasis, a complex and multifaceted process by which cancer cells spread from the primary tumor to distant organs 20. Acetate is also known to promote metastasis 17, while butyrate has the potential to suppress metastasis 21, 22.

In this study, we aimed to understand the intricate relationship between the gut microbiota, its metabolites and the progression of cancer metastasis. As the main cancer type, we chose malignant melanoma, which is the most serious type of skin cancer that originates from melanocytes. It first grows by spreading along the epidermis in the early stage of its development. Eventually, in the later stage, melanoma can invade the dermis and metastasise to distant organs, with the lungs being the most common metastatic site 23, 24. We therefore chose a mouse model of malignant melanoma lung metastasis (MM-LM) as the primary focus of our study. We aimed to identify microbial metabolites that are involved in MM-LM progression. Additionally, we also investigated the effects of these metabolites in the pancreatic ductal adenocarcinoma lung metastasis (PDAC-LM) mouse model to understand whether they can affect other types of cancers. Identification of new metabolites can potentially enable us to open new avenues for cancer prevention and treatment, and ultimately, we may pave the way for personalised cancer interventions that harness the power of the microbiome and its diverse metabolites to improve patient outcomes.

## Results

### Gut Microbiota Dysbiosis Exacerbates Melanoma Metastasis and Reduces Survival

**Figure 1:**
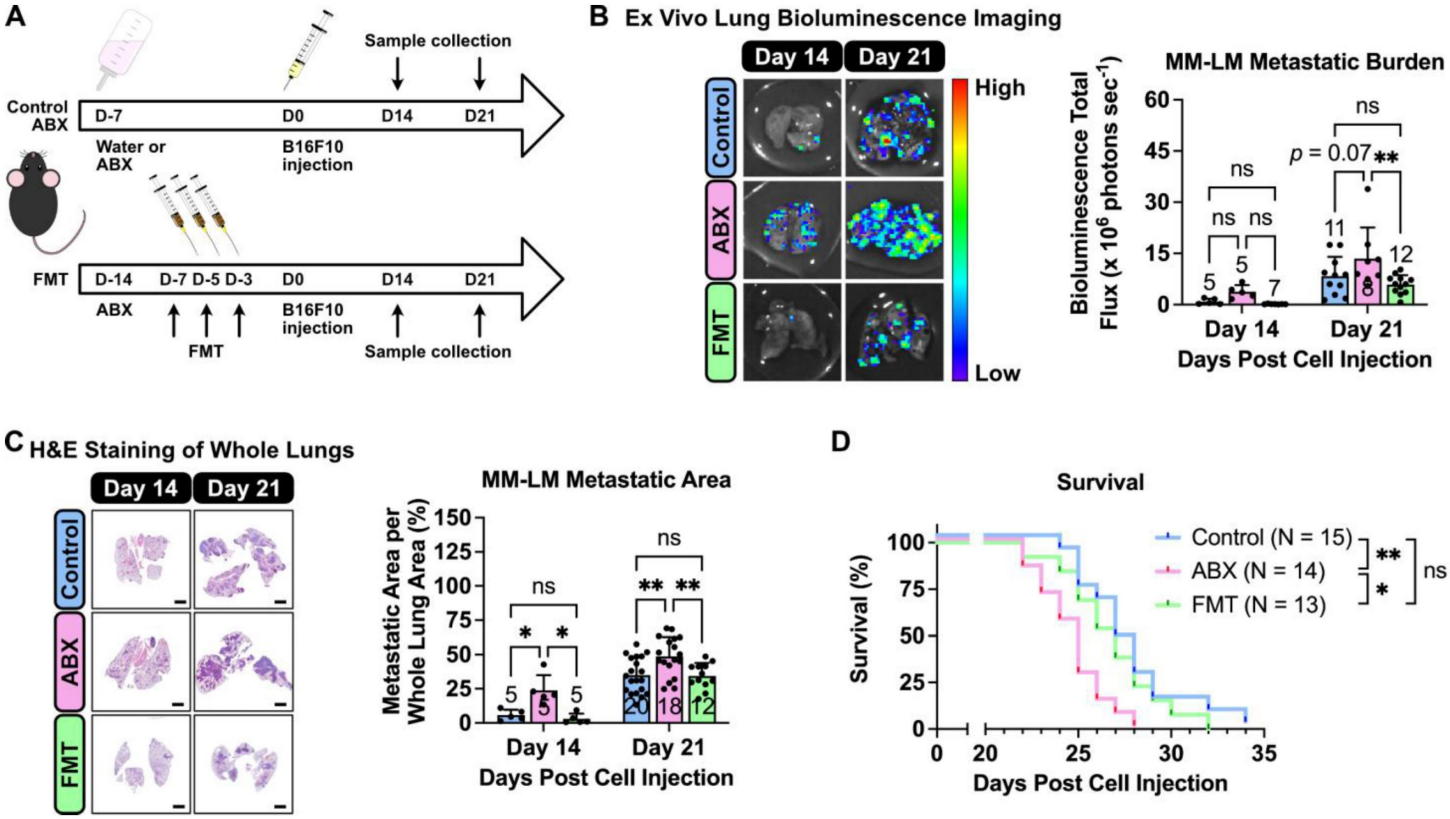
** Gut microbiota dysbiosis exacerbates melanoma lung metastasis.** (**A**) Gut microbiota dysbiosis was induced by ABX treatment prior melanoma cell injection. Restoration of microbiota population was achieved by FMT. On the day of tumor inoculation, 2 × 10^5^ B16F10 melanoma cells were injected through the tail vein. ABX treatment was continued until the end of the experiment. The mice were then sacrificed on days 14 and 21. (**B**) *Ex vivo* bioluminescence imaging was used to determine lung metastasis burden, analyzed by two-way analysis of variance with Tukey's correction. (**C**) H&E staining of excised lungs was used to determine the metastatic area in the lungs, analyzed by two-way analysis of variance with Tukey's correction. Scale bar: 2 mm. (**D**) Kaplan-Meier survival curve for all treatment groups. Pairs of survival curves were compared by log-rank (Mantel-Cox) test. ^*^P < 0.05;^ **^P < 0.01; ns, not significant.

Prior to melanoma inoculation, C57BL6/J mice underwent antibiotic (ABX) treatment to induce dysbiosis, followed by a fecal microbial transplant (FMT) to restore the gut microbial population (Figure 1A). These interventions enabled us to evaluate the influence of altered gut microbiota population on the progression of MM-LM. To monitor tumor growth, we used luciferin-expressing B16F10 melanoma cells, which were injected intravenously to induce lung metastasis. On days 14 and 21 post-inoculation, the mice were sacrificed and the lungs were excised for tumor burden quantification. Both *ex vivo* lung bioluminescence imaging (Figure 1B) and histology analysis (Figure 1C) revealed that ABX lungs bore significantly higher tumor burden than control and FMT lungs. Due to various factors that can limit bioluminescence imaging (tissue thickness, ability of D-luciferin to reach and be absorbed by the tumor *ex vivo* as well as the effect of melanin pigment in melanoma 25, histology analysis was our primary method of determination and quantification of metastatic burden. Furthermore, a survival study was also conducted (Figure 1D) and the results showed that ABX mice had significantly shorter survival rates than both control and FMT. Hence, it can be concluded gut microbiota dysbiosis exacerbates MM-LM progression, resulting in higher tumor burden and shorter survival rate, while restoring the gut microbial population through FMT can mitigate these effects.

### Gut Microbiota Dysbiosis Alters Metabolite Levels in melanoma Progression, Highlighting Formate as a Key Player

**Figure 2:**
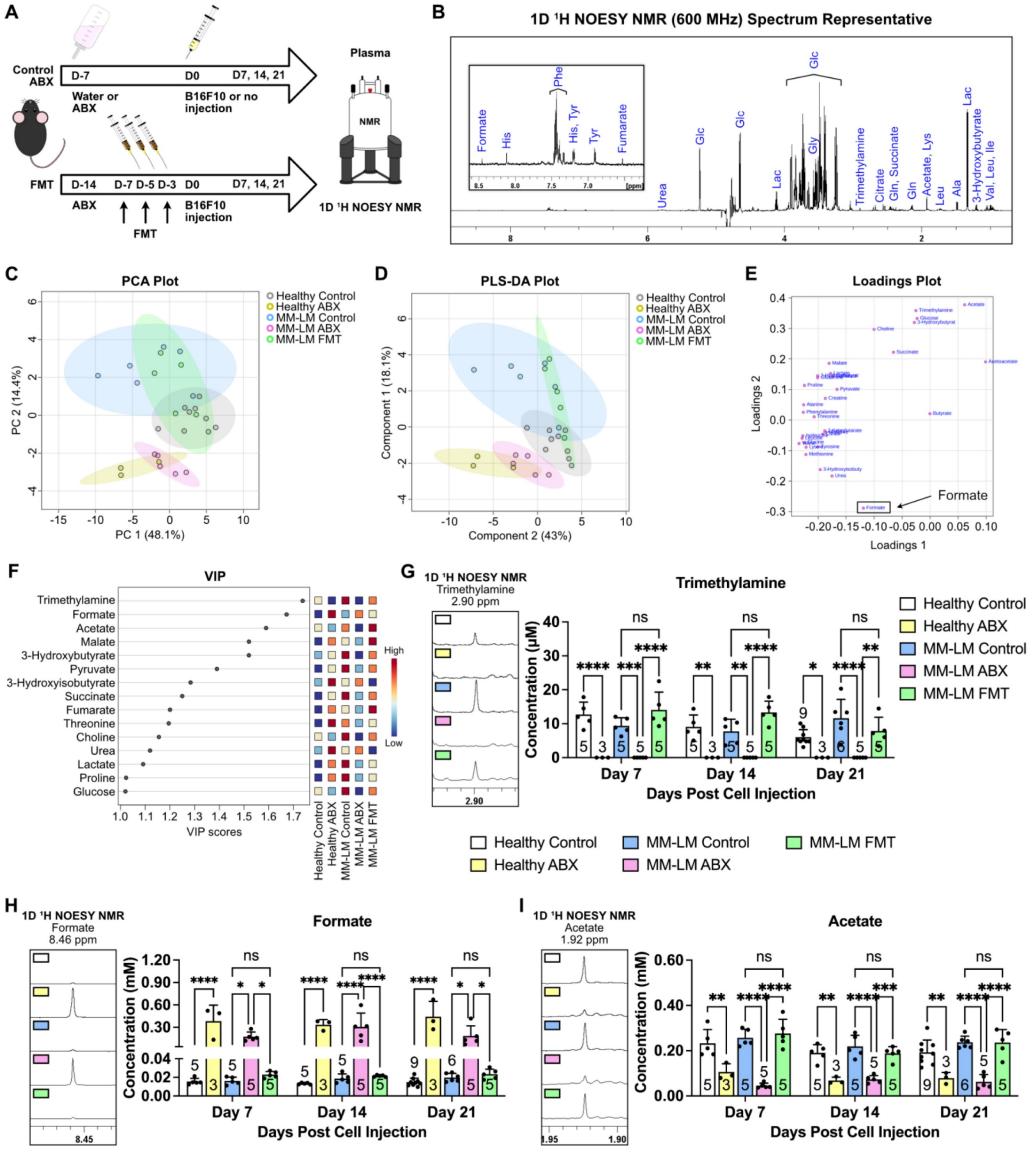
** Gut microbiota dysbiosis causes perturbation in circulating metabolites.** (**A**) Gut microbiota dysbiosis was induced by ABX treatment prior melanoma cell injection. Restoration of microbiota population was achieved by FMT. On the day of tumor inoculation, 2 × 10^5^ B16F10 melanoma cells were injected through the tail vein. Healthy (non-tumor) control and ABX mice were subjected to the same treatment without the injection of melanoma cells. ABX treatment was continued until the end of the experiment. The mice were then sacrificed on days 7, 14 and 21. Plasma and lung samples were subjected to an NMR-based metabolomics study. (**B**) Representative spectrum of 1D ^1^H NOESY NMR of a plasma sample showing identifiable metabolites. (**C**) Unsupervised PCA analysis of the five treatment groups on day 21 showed ABX-treated clusters separated from control and FMT clusters. (**D**) Supervised PLS-DA of the five treatment groups on day 21 also showed the separation of ABX-treated clusters. (**E**) Loadings plot showing formate to be the metabolite driving the separation of ABX group. (**F**) VIP plot showing trimethylamine, formate and acetate as the top three metabolites. (**G**-**I**) Weekly changes of circulating trimethylamine (**G**), formate (**H**) and acetate (**I**) concentrations in plasma across five different treatment groups over the course of 21 days treatment, analyzed by two-way analysis of variance with Tukey's correction. ^*^P < 0.05;^ **^P < 0.01; ^***^P < 0.001; ^****^P < 0.0001; ns, not significant.

We then aimed to investigate perturbations in metabolism induced by dysbiosis through nuclear magnetic resonance (NMR)-based metabolomics analysis of plasma samples 21 days after melanoma cell inoculation (Figure 2A). We chose NMR as it is highly reproducible, quantitative and non-destructive to the samples compared to mass spectrometry-based metabolomics 26. This enables us to perform further analysis to the samples whenever necessary. Using 1D Nuclear Overhauser Effect Spectroscopy (NOESY) 27 ^1^H NMR (600 MHz) with water suppression of plasma samples, we were able to confidently identify 32 metabolites (Figure 2B). Interestingly, the Principal Component Analysis (PCA) plot of plasma samples showed similarity between ABX-treated groups regardless of tumor burden (Figure 2C). Furthermore, similarity was also observed between FMT and control groups, which indicates that reintroduction of gut microbiota by FMT was able to restore metabolism perturbed by ABX-induced dysbiosis. This was further confirmed by partial least squares-discriminant analysis (PLS-DA), which showed similar trend (Figure 2D). Loadings plot revealed formate to be the metabolite as a key player that gave rise to the effects seen in the ABX groups (Figure 2E). Variable important plot (VIP) showed that formate was among the top three important metabolites among these groups, along with trimethylamine and acetate (Figure 2F). Moreover, it also showed that while trimethylamine and acetate were low in ABX-treated groups, formate was noticeably high. It is widely known that gut microbiota secretes trimethylamine 14, 28 and acetate 14, 15, 29. Therefore, ABX-induced dysbiosis was able to eliminate acetate- and trimethylamine-producing bacteria, lowering the concentration of these metabolites. We also analyzed these metabolites in the plasma 7- and 14-days post melanoma cell inoculation and found similar trends (Figures 2G-I). To validate NMR detection of formate, we tested standard SCFA mixtures. Formate was detectable at 10 μM, with no overlap from acetate, propionate, or butyrate signals (Figure S1), confirming the method's sensitivity and specificity. We also found that formate concentration in healthy control, MM-LM control and MM-LM FMT groups were similar and within the range of normal circulating formate concentration in mice, which was previously reported to be 0.01 to 0.03 mM 30, 31. Surprisingly, formate concentration increased by 10 to 25-fold in healthy ABX and MM-LM ABX plasma, suggesting the bacteria that were still present during dysbiosis by ABX treatment caused an increase in plasma formate level (Figure 2H). In addition, B16F10 melanoma cells cultured *in vitro* were found to secrete formate into the media (Figure S2), indicating that tumor cells are capable of producing formate. However, comparing between MM-LM control and ABX-treated groups, the markedly higher plasma formate levels observed in the latter suggested that gut microbiota was the dominant source *in vivo*.

### Formate Promotes Cancer Cell Proliferation, Migration, and Metastasis

**Figure 3:**
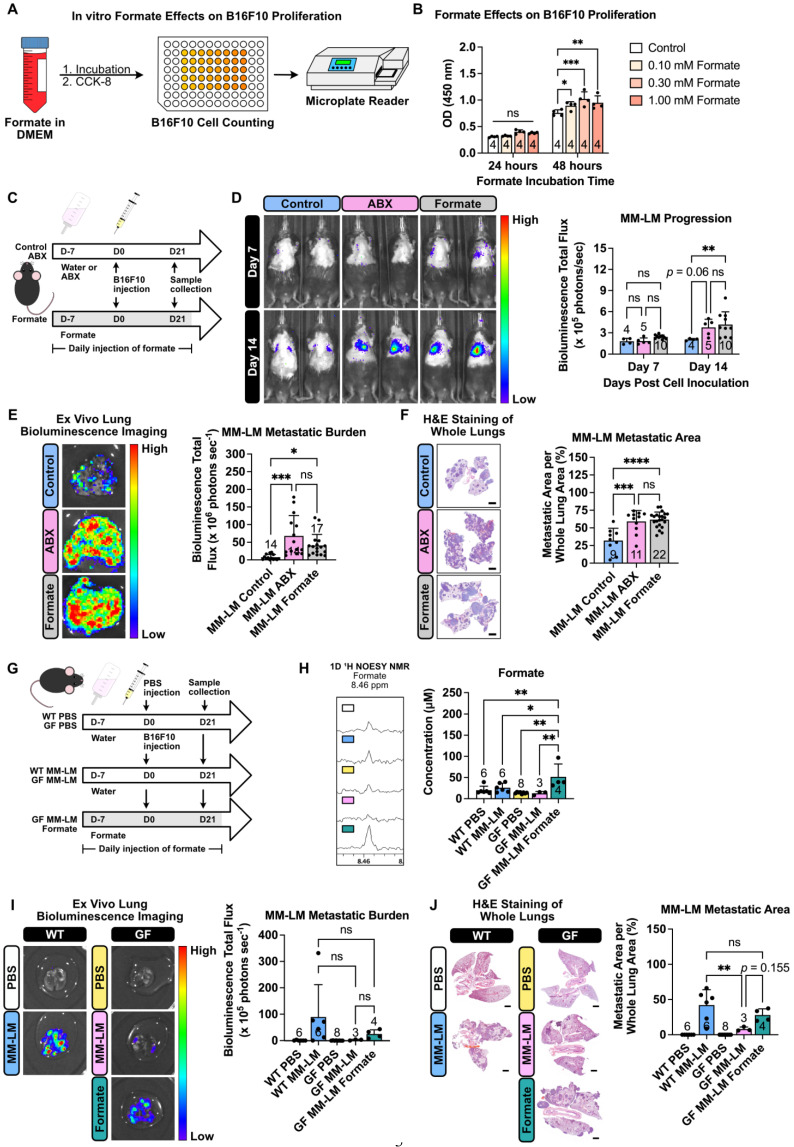
** Formate supplementation increases malignant melanoma metastasis aggressiveness.** (**A**) *In vitro* experiments studying the effects of formate on melanoma proliferation. Using a 96-well plate, 1.8 × 10^3^ B16F10 cells were incubated with formate for a period of time and the total cell number at the end of each time point was counted using the Cell Counting Kit-8 (CCK-8). (**B**) CCK-8 results showing formate ability to increase the proliferation rate of melanoma cells, analyzed by two-way analysis of variance with Dunnett's correction. (**C**) Mice were given formate supplementation to increase the amount of circulating formate prior to cell inoculation. On the day of tumor inoculation, 2 × 10^5^ B16F10 melanoma cells were injected through the tail vein. Formate supplementation and ABX treatment were continued until the end of the experiment. (**D**) IVIS was used to monitor tumor progression, analyzed by two-way analysis of variance with Tukey's correction. (**E**) *Ex vivo* bioluminescence imaging was used to determine lung metastasis burden, analyzed by one-way analysis of variance with Tukey's correction. (**F**) H&E staining of excised lungs was used to determine the metastatic area in the lungs, analyzed by one-way analysis of variance with Tukey's correction. Scale bar: 2 mm. (**G**) Germ-free mice were either kept on sterile water or supplemented daily with formate via intraperitoneal injection for 7 days before inoculation. On the day of tumor inoculation, 2 × 10^5^ B16F10 melanoma cells were injected through the tail vein. Formate supplementation was continued until the end of the experiment. (**H**) Changes of circulating formate concentrations in plasma across five different treatment groups over the course of 21 days treatment, analyzed by one-way analysis of variance with Tukey's correction. (**I**) *Ex vivo* bioluminescence imaging was used to determine lung metastasis burden, analyzed by two-way analysis of variance with Tukey's correction. (**J**) H&E staining of excised lungs was used to determine the metastatic area in the lungs, analyzed by one-way analysis of variance with Tukey's correction. Scale bar: 2 mm. ^*^P < 0.05;^ **^P < 0.01; ^***^P < 0.001; ^****^P < 0.0001; ns, not significant.

Formate, the simplest carboxylic acid with just a single carbon atom, has recently garnered attention as a significant metabolite in cancer progression. Increased formate level in cancer patients was previously reported for patients with oesophageal cancer (13-22 fold increase) 32 and rectal cancer (2 fold increase) 33, with higher concentrations detected in patients in the later stage of the disease. Most recently, formate has been shown to exacerbate colorectal cancer progression via activation of the AhR signalling pathway 34. Another study has also reported the exacerbation of Lewis lung carcinoma and B16F10 lung metastasis following ABX treatment, however, formate was not investigated in this study 35. To investigate formate influence on cancer metastasis, specifically on MM-LM progression, we performed both *in vitro* and *in vivo* investigations. *In vitro*, we first incubated B16F10 cells in a formate-containing medium with different formate concentrations for a period and quantified the cell growth using cell counting kit-8 (CCK-8) assay (Figure 3A). After 48 h, we found formate was able to enhance cell proliferation, though increasing concentrations did not further accelerate growth (Figure 3B). Additionally, we also performed wound healing assay, commonly used to measure the ability of cancer cells to migrate, which is also representative of their metastasis capability 36, 37. We observed a dose-dependent trend, whereby a higher formate dose resulted in a higher percentage of wound closure, indicating higher migration and metastatic capability (Figure S3). We then tried to study formate influence towards lung metastasis progression *in vivo* by supplementing mice with formate to increase their circulating formate level (Figure 3C). Tumor progression, monitored using an *in vivo* imaging system (IVIS), revealed that formate supplementation significantly worsened MM-LM progression, which could be seen as early as day 14 post-tumor injection (Figure 3D). This was further confirmed by *ex vivo* bioluminescence imaging (Figure 3E) and histology analysis (Figure 3F) of day 21 lung samples.

Since formate has been shown to influence different types of cancer 32-34, we endeavour to determine whether it can also influence the metastatic capability of other cancers in the lung. To explore this, we chose PDAC, which is known to be able to metastasise to the liver and the lungs 38. Similarly to our MM-LM study, we tried to study the influence of dysbiosis and formate supplementation towards PDAC-LM progression *in vivo* by supplementing mice with ABX and formate to increase their circulating formate level (Figure S4A). IVIS used to monitor tumor progression confirmed that ABX-induced dysbiosis and formate supplementation exacerbated PDAC-LM progression (Figure S4B). This was further confirmed by *ex vivo* bioluminescence imaging (Figure S4C) and histology analysis (Figure S4D) of lung samples on day 21. Since these findings align with those observed in the MM-LM model, it can be concluded that increased circulating formate levels can exacerbate cancer metastasis capability in the lungs.

### Formate Supplementation Promotes Lung Metastasis in Germ-Free Mice

To further isolate the effects of gut microbiota-derived formate induced by dysbiosis on lung metastasis, we utilized germ-free (GF) mice. To increase the circulating formate levels, GF mice were supplemented with formate for 7 days prior to B16F10 melanoma cell injection via the tail vein and continued throughout the experiment (Figure 3G). Comparing conventional wild type (WT) and GF groups, we saw a similar level of plasma formate (Figure 3H). This was expected, as we hypothesised that the increased plasma formate level in dysbiotic mice originated from the bacteria that were still present after ABX treatment. Compared to GF control, we observed a significant increase of circulating formate levels in the formate-supplemented GF MM-LM group (Figure 3H). Comparing conventional WT MM-LM with GF MM-LM, we saw a lower level of metastatic burden in GF MM-LM despite having a similar plasma formate level. Simultaneously, formate-supplemented GF MM-LM mice showed increased lung metastasis burden compared to non-supplemented GF MM-LM (Figure 3I-J). These results provide evidence that elevated formate alone is sufficient to exacerbate lung metastasis. This is in agreement with our previous observations of ABX-treated MM-LM and PDAC-LM models. Taken together with the previous results, we observed that dysbiosis-induced perturbation of gut microbiota resulted in increased plasma formate levels, which in turn became a key factor contributing to heightened tumor metastatic potential. Our findings in GF mice provided strong support to confirm the role of gut-microbiota-derived formate in our dysbiotic metastasis models. However, it is important to interpret these results in the context of known physiological differences in GF mice, such as exhibiting widespread immune immaturity, reduced baseline inflammation, altered cytokine profiles, and differences in vascular integrity and tissue architecture 39-41. These features may influence tumor cell extravasation, immune evasion, and metastatic outgrowth following intravenous inoculation. With these known physiological traits, we also observed that the success rate of melanoma lung metastasis induction in GF mice was lower than in WT counterparts, despite using the same inoculation protocol. This likely reflects the distinct tumor-host interactions in the GF setting and highlights the challenges of comparing metastatic efficiency across these models. Despite these limitations, our findings in GF mice provided strong support for a causal role of formate in metastasis, while also emphasising the need for careful contextual interpretation when using GF models to evaluate cancer progression.

### *Enterobacterales* Dominates Gut Microbiota Post-ABX Treatment Linked to Formate Production

**Figure 4:**
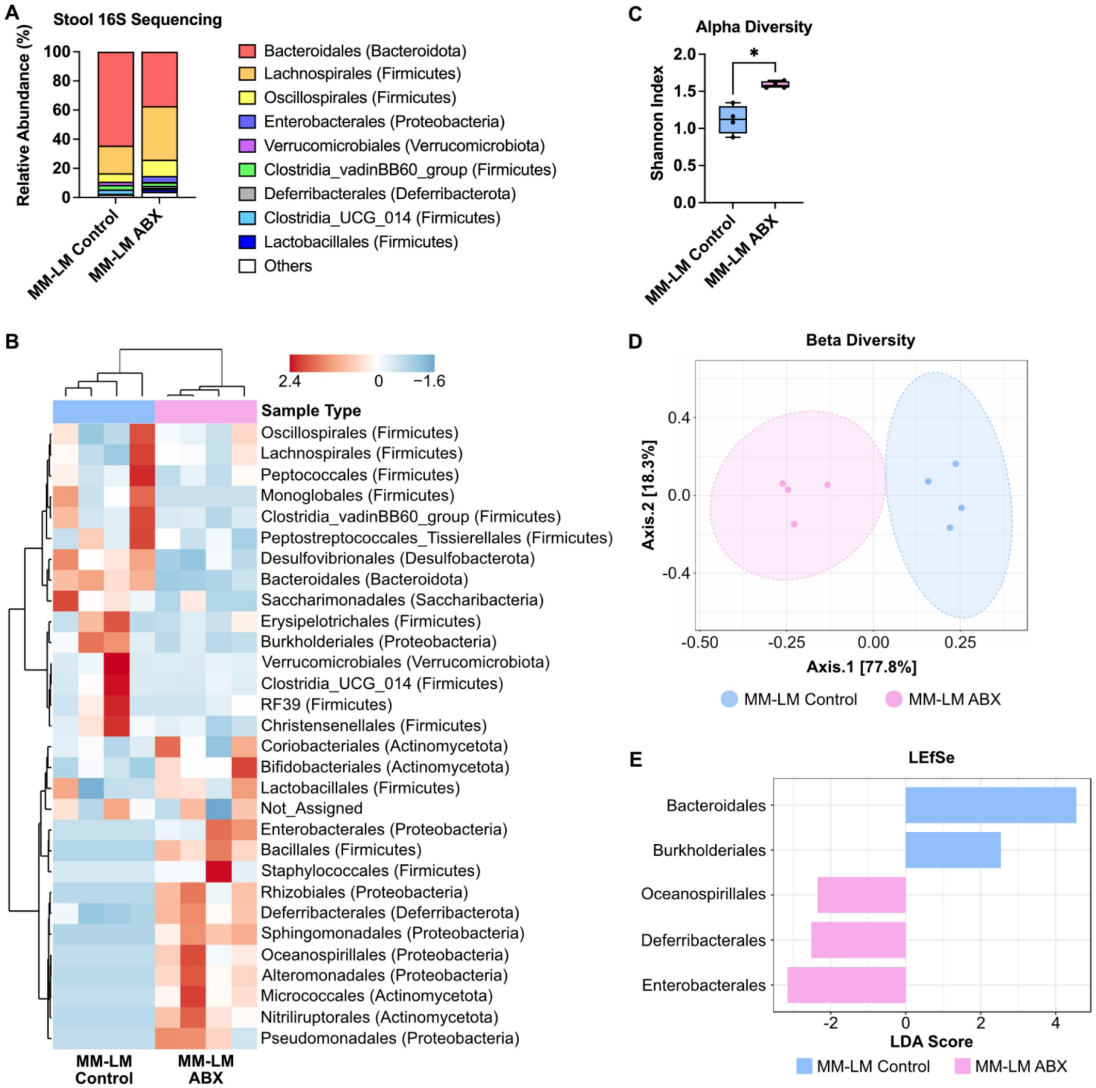
** ABX treatment increases the population of pathogenic gut bacteria.** (**A**) Relative abundance of fecal bacteria population at the order level. (**B**) Heatmap cluster analysis of fecal bacteria. (**C**) Shannon alpha diversity analysis of fecal bacteria, analyzed by *t*-test. (**D**) Beta diversity analysis of fecal bacteria. (**E**) LEfSe analysis of stool bacteria reveals *Enterobacterales* as the most dominant bacteria in ABX-treated group. ^*^P < 0.05.

A previous study shows that formate is of microbial origin, produced in the intestinal lumen and is detected in a low concentration in the intestines of germ-free mice 42. To proof our hypothesis that increased plasma formate came from the bacteria that were still present after ABX treatment in dysbiotic mice. We endeavoured to identify these bacteria using next-generation 16S sequencing of stool bacteria. It is usually assumed that ABX treatment would eradicate the gut microbiome population, however our results showed changes in bacterial composition after ABX treatment, indicating that not all bacteria species were eliminated by ABX treatment (Figure 4A-B). This is expected as there are also several published studies reporting the presence of leftover bacteria after ABX treatment 35, 43. Alpha and beta diversity analysis both showed changes in ABX-treated bacteria diversity (Figure 4C-D). LEfSe analysis revealed *Enterobacterales* as the most dominant bacteria in the ABX-treated group (Figure 4E). The dominance of *Enterobacterales* was statistically supported by linear discriminant analysis effect size (LEfSe), which revealed a significant enrichment in ABX-treated mice (LDA score -3.15, FDR-adjusted *p* = 0.042). Although other microbial phyla such as Firmicutes and Bacteroidetes are known to produce SCFAs, they were substantially depleted post-ABX, and no other taxa showed significant enrichment that correlated with increased plasma formate. While 16S rRNA sequencing enabled us to detect major shifts in microbial composition following antibiotic treatment, it is inherently limited in both taxonomic resolution and the ability to infer functional capacity or absolute abundance of individual taxa. Our 16S rRNA sequencing analysis identified *Enterobacterales* as the predominant bacterial order retained after antibiotic treatment (Figure 4E), consistent with previous studies reporting their capacity to produce formate via pyruvate-formate lyase (PFL) under anaerobic conditions 44. This compositional shift suggests that *Enterobacterales* may serve as a major microbial source of circulating formate in dysbiotic mice.

### Gene Expression Analysis of B16F10 Melanoma Cells Post-Formate Supplementation

Given the metastatic-promoting effect of formate in our formate-supplemented and dysbiosis lung metastasis models, and its role in the one-carbon metabolism pathway (Figure S6A), we sought to understand the underlying mechanism of how formate supplementation affects the gene expression profile of B16F10 melanoma cells. We performed a comprehensive gene expression analysis of one-carbon metabolism pathway after incubating the cells in formate-supplemented DMEM for 48 h (Figure S6B). The analysis revealed significant changes in the expression of genes related to one-carbon metabolism. Key regulatory genes, including those involved in purine synthesis, exhibited marked upregulation (Figure S6C-N), indicating an enhanced metabolic response to increased formate availability. Of note, methylenetetrahydrofolate dehydrogenase 2 (*Mthfd2*) gene was significantly upregulated (Figure S6I). MTHFD2 is an enzyme that is expressed in embryos but not in healthy adult tissues 45. It is also expressed in many types of cancers and can aid cancers in evading the immune system 46 and thus possibly supporting metastasis. These findings support the notion that formate directly influences the metabolic pathways within melanoma cells, potentially contributing to increased tumor aggressiveness and metastatic capability.

### Formate Fuels Nucleotide Synthesis in Melanoma Cells Through One-Carbon Metabolism

**Figure 5:**
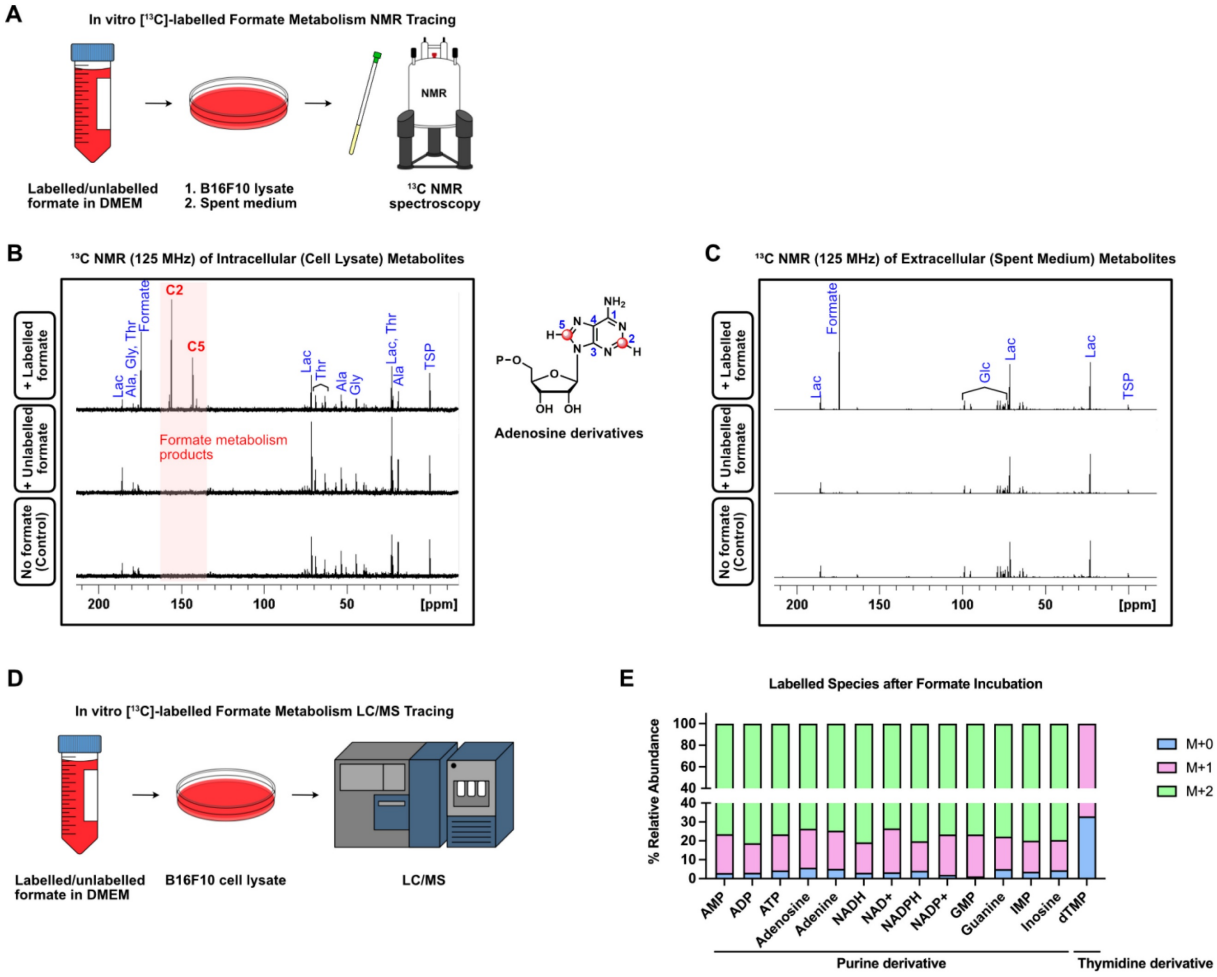
** Formate is incorporated into purine-derivatives by B16F10.** (**A**) B16F10 cells were incubated with ^13^C-labelled and unlabelled formate and subjected to ^13^C NMR. (**B**) ^13^C NMR (125 MHz) spectra of intracellular (cell lysate) metabolites of B16F10 incubated with labelled formate (top), unlabelled formate (middle) as well as no formate (bottom). (**C**) ^13^C NMR (125 MHz) spectra of extracellular (spent medium) metabolites with labelled formate (top), unlabelled formate (middle) as well as no formate (bottom). (**D**) LC/MS-based metabolomics of B16F10 cell lysate was used to trace the fate of ^13^C-labelled formate. (**E**) Labelled species detected by LC/MS after formate induction.

Formate and one-carbon metabolism are known to be involved in the synthesis of purine nucleotides, synthesis of thymidylate and provision of methyl groups to *S*-adenosylmethionine (SAM) 47. Rapidly proliferating cells, such as cancer cells, have an increased need for nucleotide synthesis, which can be fulfilled by formate via one-carbon metabolism (Figure 5A) 30. To understand how formate affects melanoma cell nucleotide synthesis, we incubated B16F10 cells in both ^13^C-labelled and unlabelled (^12^C) formate-containing DMEM, with additional control cells incubated in DMEM without formate. After 48 h of incubation, the cells and the spent medium were collected and subjected to ^13^C NMR spectroscopy for analysis of intracellular and extracellular metabolites, respectively (Figure 5B-C). The ^13^C NMR spectra of intracellular metabolites from cell lysate showed several ^13^C peaks around δ 140-160 ppm in the cells incubated in ^13^C-labelled formate/DMEM that were not observed in the cells incubated in unlabelled formate/DMEM and DMEM only. This indicates that the peaks originated from metabolites that incorporated ^13^C from formate into their structures (Figure 5B). In contrast, the ^13^C NMR spectra of extracellular metabolites did not show any remarkable peaks (Figure 5C), suggesting that none of the ^13^C-labelled metabolites were excreted by the cells. Further analysis using 2D NMR (COSY and HSQC) showed that the cluster of peaks was likely to be adenosine derivatives. Due to their structural similarity, NMR spectroscopy was not suitable to differentiate and identify the metabolites that gave rise to those peaks. Nevertheless, liquid chromatography/mass spectrometry (LC/MS) can differentiate between these metabolites based on their corresponding mass/charge (*m/z*) values. Hence, to identify formate-derived metabolites, we employed LC/MS-based metabolomics of B16F10 cells incubated in ^13^C-Formate/DMEM and ^12^C-Formate/DMEM (Figure 5D). Depending on the structure and metabolic pathway, the metabolic products may incorporate one or more ^13^C that originated from ^13^C-formate. Therefore, these metabolites would give rise to [M+1] and [M+2] species in the mass spectrum. We identified 14 metabolites, consisting of 13 purine derivatives and a thymidine derivative (dTMP), which are consistent with the findings from ^13^C NMR (Figure 5E). Overall, we confirmed that in melanoma cells, formate is actively utilised in nucleotide synthesis.

### Formate Supplementation Induces Significant Metabolic Changes and Enhances Metastatic Potential in B16F10 Melanoma Cells

**Figure 6:**
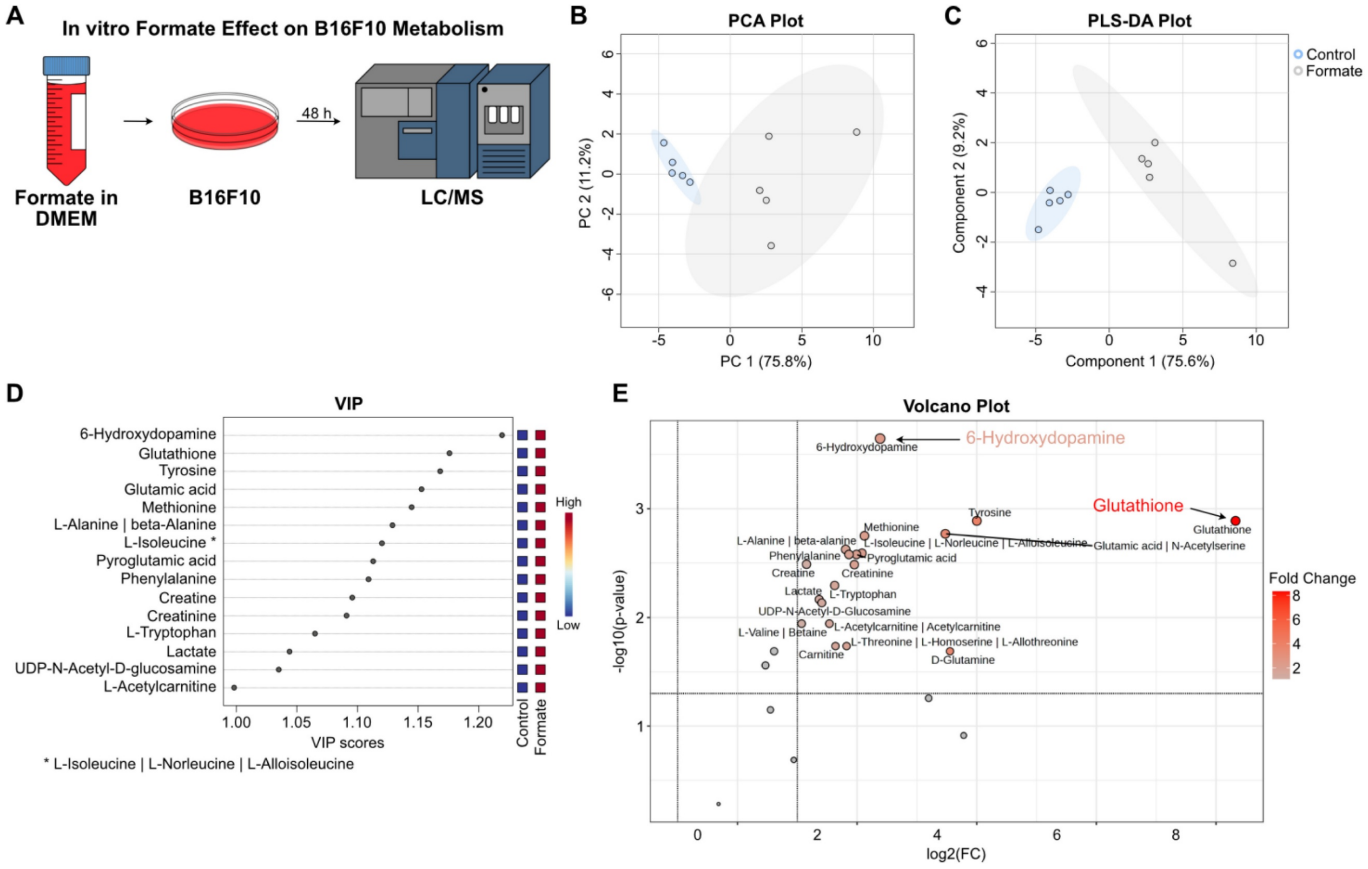
** Metabolic changes in B16F10 melanoma cells post formate supplementation.** (**A**) Workflow of the experiment: B16F10 melanoma cells were incubated in formate-supplemented DMEM for 48 h, followed by LC/MS analysis of the cell lysates. (**B**) Unsupervised PCA analysis of B16F10 cells metabolites showed a separation between the two groups. (**C**) Supervised PLS-DA analysis of B16F10 cells metabolites also showed a separation between the two groups. (**D**) VIP plot showed the top 15 metabolites of B16F10 cells significantly altered by formate supplementation. (**E**) Volcano plot illustrating significant changes in metabolite levels, with GSH and 6-OHDA showing the most substantial differences (adjusted *p* values: 0.00023 and 0.00129, respectively).

Even though formate is directly related to purine synthesis, we were also interested to see how it changes the metabolome of the melanoma cells post-exposure. Hence, to investigate the effects of formate on B16F10 metabolism, we incubated the cells in formate-supplemented DMEM for 48 h and analyzed the lysate by LC/MS to capture the metabolic alterations induced by formate exposure. (Figure 6A). PCA showed that the metabolic profile was changed following formate supplementation (Figure 6B). This observation was further validated by PLS-DA, which demonstrated a clear separation between the formate-supplemented group and the control group, indicating significant metabolic changes (Figure 6C). VIP analysis showed 6-hydroxydopamine (6-OHDA) and glutathione (GSH) as the top two significantly altered metabolites, followed by several amino acids (Figure 6D). The volcano plot showed that while 6-OHDA and GSH were significantly changed in the formate group (*p* adjusted with FDR = 0.00023 and 0.00129, respectively), it should be noted that GSH was produced at a much higher level than 6-OHDA (320.98 vs 5.22, respectively) in formate group (Figure 6E). 6-OHDA, known as a neurotoxin, has been shown in mouse models to slow the progression of neuroblastoma and breast adenocarcinoma but not melanoma 48. It is not surprising for melanoma to produce a neurotoxin, since a lot of solid tumors, including melanomas, are innervated 49. GSH, on the other hand, is known to be closely related to cancers. It is an antioxidant with a multitude of functions, but it mainly helps to maintain redox homeostasis in cells. Excess levels of GSH are known to promote tumor progression and metastasis 50. Therefore, we believe that formate supplementation resulted in the aggravation of lung metastasis, due to the increased production of GSH.

## Discussion

Gut microbiota dysbiosis, characterised by an imbalance in the microbial community, has been increasingly associated with various cancer types, including colorectal 4, 5, liver 7, 8, and breast cancers 12, 13. In our study, we observed that ABX-induced dysbiosis significantly exacerbated melanoma lung metastasis in a mouse model. This finding is consistent with previous reports indicating that dysbiosis can promote tumor progression and metastasis by altering the systemic environment 42. The restoration of gut microbiota through fecal microbiota transplantation (FMT) mitigated these effects (Figure 1), suggesting that maintaining a healthy microbiome is critical in preventing metastasis and improving cancer outcomes.

Formate, an SCFA and a product of microbial metabolism 34, emerged as a key player in our study (Figure 2). Elevated plasma formate levels in ABX-treated mice were associated with an increased metastatic burden, particularly in the lungs, which aligns with previous research showing that formate is present at higher concentrations in cancer patients 32, 33 and plays a role in cancer progression through pathways such as one-carbon metabolism and nucleotide biosynthesis 30, 47. Our findings extend these observations by demonstrating that formate can enhance the aggressiveness of melanoma and PDAC lung metastasis (Figure 3), suggesting that it could be a potential target for therapeutic interventions. To outline the role of microbiota-derived formate, we performed complementary experiments in germ-free (GF) mice as these mice inherently lack gut microbiota and were never exposed to antibiotics. Formate supplementation in GF mice led to a marked increase in lung metastasis compared to GF controls, confirming that elevated circulating formate is sufficient to promote pulmonary colonisation by tumor cells, independent of antibiotic-induced confounding effects (Figure 3).

This was further confirmed through *in vitro* experiments, where formate supplementation was shown to promote the proliferation and migration of melanoma cells, key processes involved in metastasis (Figure 3 and Figure S3). The dose-dependent increase in wound closure observed in the wound healing assay indicates that formate not only supports cell proliferation but also enhances the migratory capacity of cancer cells, further contributing to their metastatic potential. Notably, this proliferative effect reached a plateau at concentrations near 1 mM, suggesting saturation of formate-utilising pathways without inducing apparent cytotoxicity. Although we did not observe inhibitory effects within the tested range, future studies are needed to determine whether excessively high levels of formate may impair cell viability or trigger metabolic stress responses.

The findings from this study add to the growing body of evidence that gut microbiota and its metabolites are integral to cancer progression and metastasis. The microbiome analysis showed that ABX treatment resulted in a significant shift in the gut microbiota composition, with *Enterobacterales* emerging as the dominant bacterial group in dysbiotic mice. This change in bacterial population could explain the increased production of formate, as certain bacterial taxa within *Enterobacterales* are known to produce formate as a metabolic byproduct 42 (Figure 4). The persistence of these bacteria despite ABX treatment underscores the resilience of certain pathogenic bacterial populations and highlights the need for more targeted approaches in microbiome modulation, especially in cancer patients who are at risk of metastasis.

Gene expression and metabolic profiling of B16F10 melanoma cells post-formate supplementation revealed significant alterations that highlight the profound impact of formate on the cells' metabolic landscape and their aggressive phenotype. The gene expression analysis showed a notable upregulation of genes involved in one-carbon metabolism, particularly those related to purine and nucleotide biosynthesis, suggesting an enhanced metabolic capacity that may contribute to increased proliferation and metastatic potential (Figure 5). Other studies have shown formate supplementation can induce a metabolic switch, elevating high adenine nucleotide levels via one-carbon metabolism, increasing glycolysis rate and lowering AMPK activity in colorectal cancer 47. Additionally, another study reported that formate is involved in lipid metabolism through fatty acid synthesis, which increases cancer invasiveness 51. These studies show that these pathways are metabolically interconnected with each other and suggest that formate plays several roles in multiple key metabolic processes in cancers. Additionally, formate supplementation may also affect tumor microenvironment, immune cells metabolism and immune invasion. A study showed that formate can improve the outcome of cancer immunotherapy by enhancing CD8+ T-cells and the efficacy of PD-1 blockade 52. At the same time, there are also studies that show formate can exacerbate cancer progression 34, increase the invasiveness of cancer cells and promote metastasis 51, all of which are in line with our results. Together with our results, all of these studies indicate that formate play a multifaceted role in cancer progression and metastasis and that future studies should focus endeavour to establish these intricate relationships, especially in the context of pulmonary metastasis.

Metabolic profiling of formate-supplemented B16F10 cells uncovered a significant increase in the production of 6-OHDA and GSH, metabolites associated with neurotoxin activity 48 and redox homeostasis 50 respectively (Figure 6). The presence of 6-OHDA suggests a potential, though not yet fully understood, role in tumor cell interactions with the nervous system 49. Future studies could explore other potential roles of 6-OHDA in melanoma, such as its involvement in tumor innervation and metastatic progression. Although much is still yet to be elucidated, GSH is well known to play a role in cancer initiation, progression and metastasis 50. While GSH is important in the clearance of carcinogens, elevated levels of GSH in tumor cells can exhibit protective effects towards these cells in several organs including the lungs 50. It has also been shown to promote melanoma metastasis 50, 53, which supports our overall findings.

While our study provides significant insights into the role of gut microbiota-derived formate in exacerbating pulmonary metastasis, several limitations warrant consideration. Firstly, the investigation into the mechanism by which knocking down MTHFD2 followed by formate supplementation impacts cancer progression is an area that requires further exploration. Although our findings suggest that formate influences one-carbon metabolism and nucleotide synthesis via MTHFD2, the precise molecular pathways remain to be fully elucidated. Other than one carbon metabolism, formate transport has been associated with members of the solute carrier (SLC) family, particularly SLC16A1 (MCT1) and SLC5A8, both of which are capable of transporting monocarboxylates including formate 54, 55. However, whether these or other transporters are upregulated in melanoma cells upon formate exposure was not directly investigated in this study. Future studies could explore the expression and activity of candidate transporters through transcriptomic analysis, pharmacologic inhibition, or gene silencing approaches to determine their contribution to formate uptake and metabolic reprogramming. Expanding our understanding of the transport mechanisms and formate-mediated MTHFD2 in nucleotide synthesis may further refine therapeutic strategies targeting formate metabolism in metastatic cancers. Secondly, our study highlights the association between dysbiosis and elevated formate levels; however, we have not provided direct proof that post-antibiotic treatment gut bacteria can secrete formate. Furthermore, 16S rRNA sequencing provides limited taxonomic and functional resolution or absolute quantification of individual microbial taxa. While we identified *Enterobacterales* as the dominant bacterial group after antibiotic treatment, future investigations should incorporate quantitative PCR (qPCR) or colony-forming unit (CFU) assays to validate absolute abundance, combined with metagenomic or metatranscriptomic profiling to identify formate biosynthetic genes such as *pflB* or *fdhF* 56, 57. In addition, *in vitro* culture and metabolite quantification of *Enterobacterales* strains isolated from ABX-treated mice could help determine their actual formate production capacity. These complementary approaches will be essential to validate the mechanistic link between *Enterobacterales* expansion and systemic formate elevation in the context of dysbiosis-associated metastasis.

In conclusion, our study underscores the critical role of gut microbiota-derived formate in exacerbating the metastatic progression of melanoma and pancreatic cancers. Our findings suggest that targeting microbial metabolites like formate, or modulating the gut microbiome, could provide novel therapeutic strategies for managing metastatic cancer. Dysbiosis induced by ABX treatment was shown to elevate plasma formate levels, which contributed to the aggressive metastatic behaviour observed in these cancers. As the shortest chain member of the SCFA family, formate has garnered attention for its significant impact on cancer progression, being linked to cellular metabolism, epigenetic modifications, and oxidative stress, factors that are crucial in carcinogenesis. Many aspects of formate effects on cancer metastasis are still unknown and should be addressed in future studies. These include its influence on metabolism (e.g., lipid metabolism, glycolysis, oxidative phosphorylation), the immune system (e.g., metabolic activity of immune cells, immune evasion) and tumor microenvironment (e.g., in poorly vascularised tumors).

While there is accumulating evidence that gut microbiota can influence various aspects of cancer development, including tumor growth, immune response, and treatment efficacy, our understanding of how gut microbiota modulates the metastatic process remains limited. The interplay between gut microbiota and cancer metastasis is complex, involving intricate signalling networks, immune responses, and microenvironmental factors. Further research is needed to fully elucidate the mechanisms by which formate and other microbial metabolites influence cancer progression and to explore potential strategies for targeting the gut microbiota to prevent or suppress metastatic spread. In-depth investigations in this area may offer novel insights into the development of targeted therapies that can effectively address cancer metastasis and improve patient outcomes.

## Material and methods

### Animal Experiments

Male 10-week-old WT and germ-free C57BL/6J mice were purchased from the National Laboratory Animal Center, Taiwan and were used for all experiments. The mice were housed under a 12-hour day-night cycle with unlimited access to food and water. All animal experiments have been approved by Academia Sinica Institutional Animal Care and Use Committee (IACUC).

### Melanoma Lung Metastasis Model

#### Culture and Inoculation

Luciferase- and GFP-expressing B16F10 (B16F10-Luc-GFP) melanoma cells were cultured in Dulbecco's Modified Eagle Medium (DMEM) high glucose medium supplemented with 10% FBS. On the day of inoculation, 2 × 10^5^ cells suspended in 100 μL sterile PBS were injected into the tail vein of 12-week-old C57BL/6 mice.

#### *In Vivo* Monitoring

Tumor growth was monitored weekly through IVIS (PerkinElmer) starting from day 7 post injection. Mice were anesthetised through isoflurane inhalation and intraperitoneally injected with D-luciferin firefly potassium salt solution (200 μL, 15 mg/mL in PBS, Biosynth). A series of IVIS images was then captured (exposure time 120 s, F/stop 1) until the bioluminescence intensity of each mouse reached the maxima. Body weight was monitored weekly starting on the day of cell injection throughout the experiment.

#### *Ex Vivo* Tumor Burden Measurement

On day 14 and 21, mice were sacrificed and plasma samples were collected via cardiac puncture for NMR metabolomics study. Lungs were excised and imaged by bioluminescence imaging to quantify tumor burden. The lungs were placed on a dish and D-luciferin firefly potassium salt solution (1 mL, 300 μg/mL in PBS, Biosynth) was added to cover the whole lungs. A series of IVIS images was then captured (exposure time 60 s, F/stop 4) until the bioluminescence intensity of each lung sample reached the maxima. The lungs were then preserved in 4% paraformaldehyde, sectioned (5 μm) and subjected to H&E staining for tumor burden quantification. The final number of biological replicates per group reflects the number of mice that successfully developed detectable lung metastases following tail vein injection. Mice without observable tumor nodules in excised lung tissues judged by H&E staining were excluded from analysis. These discrepancies were not treatment-related but resulted from the natural variability of tumor engraftment in metastatic models.

### Plasma Sample Preparation for NMR Metabolomics

Whole blood was collected *via* cardiac puncture during sacrifice into heparin-containing tubes to prevent coagulation and centrifuged (3000 rpm, 20 min, 4 °C). The plasma was immediately collected and frozen in liquid nitrogen before storage at -80 °C. On the day of the analysis, Amicon ultracentrifugation tubes (MWCO 3 kDa) were washed three times with sterile ddH_2_O. Plasma samples (500 μL) were then filtered through the tubes by centrifugation (15000 rpm, 2 h, 4 °C) and the supernatant (350 μL) was collected. Into the samples were added phosphate buffer solution (100 mM) in D_2_O containing TSP (60 μM). More phosphate buffer in D_2_O was then added to achieve a final volume of 600 μL (final TSP concentration 10 μM). Samples were then transferred to 5 mm NMR tubes and the NMR spectrum was recorded using a Bruker AVIII 600 MHz NMR Spectrometer.

### B16F10 Formate Incubation and Preparation for CCK-8 Assay

B16F10 cells (9 × 10^3^ cells/mL) were seeded into 96 well plates (200 μL) and left overnight. The medium was then removed carefully without disturbing the attached cells and new medium with increasing formate concentrations (200 μL) was added. After 24 and 48 h incubation, CCK-8 (20 μL) was added and the mixture was incubated for another 2 h. Absorbance at 450 nm was then measured using a microplate reader.

### B16F10 Formate Incubation and Preparation for Wound Healing Assay

B16F10 cells (5 × 10^5^ cells per well) were seeded in 6-well tissue culture plates. After an overnight incubation, the spent medium was removed, a straight scratch (wound) was created in the monolayer using a sterile 200 µL pipette tip. Care was taken to apply consistent pressure and maintain a uniform wound width across all wells. Cells were gently washed twice with sterile phosphate-buffered saline (PBS) to remove debris and non-adherent cells. Fresh DMEM containing 0, 0.1, 0.3 and 1 mM formate was added to the wells. Wound closure was monitored by taking images of the scratch area at 0 and 24 h using an inverted phase-contrast microscope. The width of the wound at each time point was measured using ImageJ software. The percentage of wound closure was calculated by comparing the initial wound width to the width at subsequent time point.

### B16F10 Formate Incubation and Preparation for ^13^C NMR Analysis

B16F10 cells (2 × 10^6^ cells) were seeded in a 15 cm dish. After an overnight incubation, the spent medium was removed, cells were washed with PBS and new medium (25 mL) with ^13^C-labelled formate, unlabelled formate or without formate supplementation was added.

For the preparation of intracellular (cell lysate) metabolite analysis, methanol and chloroform were pre-chilled to -20 °C, while water and PBS were pre-chilled to 4 °C. After 48 h formate incubation, spent medium was taken and kept in -80 °C for extracellular metabolite analysis. Cells were then washed three times with cold PBS and cold methanol (3 mL) was added. Cells were detached using cell scrapers and transferred to a Falcon tube and put on ice. Cold chloroform (1 mL) was added and the mixture was vortexed for 30 s. More cold chloroform (1 mL) and cold water (1.5 mL) were added and the mixture was vortexed for 30 s, sonicated for 1 min and centrifuged at 4 °C, 3000 rpm, 10 min. The aqueous phase (3.5 mL) was carefully transferred into a new falcon tube and freeze-dried overnight. Dry samples were kept in -80 °C until analysis.

For the preparation of extracellular (spent medium) metabolite analysis, an Amicon ultracentrifugation tube (MWCO = 3 kDa) was washed three times with ddH_2_O (500 μL) to remove glycerol by centrifugation (4 °C, 15000 rpm, 5 min). A new collection tube was used and spent medium (500 μL) was centrifuged (4 °C, 15000 rpm, 45 min). The filtrate (300 μL) was transferred to an Eppendorf tube and freeze-dried overnight. Dry samples were kept in -80 °C until analysis.

NMR buffer containing PBS (100 mM, pH 7.4) in 100% D_2_O (5 mL) and TSP-*d*_4_ (1 mM, 0.8613 mg) was prepared. On the day of analysis, samples were dissolved in the NMR buffer (400 μL) and centrifuged (4 °C, 15000 rpm, 3 min) to remove any debris. The solution (300 μL) was carefully taken without disturbing the debris pellet and transferred into a Shigemi tube and used for NMR analysis.

### B16F10 Formate Incubation and Preparation for LC/MS Formate ^13^C Tracing Metabolomics

B16F10 cells (1 × 10^6^ cells) were seeded in a 10 cm dish. After an overnight incubation, the spent medium was removed, and the cells were washed with warm PBS and new medium (10 mL) with and without ^13^C-labelled/unlabelled formate was added. After 48 h of incubation, the cells were washed three times with cold PBS, detached using cell scrapers and the suspension was centrifuged (4 °C, 200 rpm, 5 min). Following the removal of PBS, 500 μL ice MeOH was added. The mixture was vortex with GenoGrinder at 1000 rpm for 2 min and then put on ice for 5 min. Then, adding 500 μL ice water and vortex with GenoGrinder at 1000 rpm for 2 min and then put on ice for 5 min. Centrifuge at 15,000 rpm for 5 min. The supernatant was carefully taken and freeze-dried. Dried samples were immediately sent to the NTU Centers of Genomic and Precision Medicine, Taiwan, for LC/MS measurement.

### ^1^H NMR Spectroscopy for Metabolomics Study

All ¹H NMR spectra were recorded at the High Field Nuclear Magnetic Resonance Center, Academia Sinica, Taiwan, using a Bruker AVIII 600 MHz NMR spectrometer. The spectra were acquired using the standard 1D ¹H NOESY pulse sequence with water suppression (128 scans, 64k data points). After acquisition, the FID was zero-filled to 128k data points and Fourier transformed with an exponential line broadening of 0.3 Hz. Phase and baseline corrections were applied prior to analysis. Metabolite peaks were assigned based on data from the Human Metabolome Database (HMDB) and Biological Magnetic Resonance Bank (BMRB), with additional verification using 2D NMR techniques such as COSY, HSQC, and HMBC.

### ^13^C NMR Spectroscopy for Formate Tracing Experiment

All ^13^C NMR spectra were recorded at the High Field Nuclear Magnetic Resonance Center, Academia Sinica, Taiwan, using a Bruker Avance 500 MHz NMR spectrometer. The spectra were acquired with the standard Bruker *zgpg30* pulse sequence (9,000 scans, 64k points). After acquisition, the FID was zero-filled (128k points) and Fourier transformed with an exponential line broadening (1.0 Hz). Phase and baseline corrections were applied to the spectra before analysis. Peaks were assigned according to previously-published data 58, the human metabolome database (HMDB) and Biological Magnetic Resonance Bank (BMRB), with further confirmation through 2D TOCSY, HSQC and HMBC experiments.

### Software

IVIS images of tumor bioluminescence were collected and processed using Living Image 3.1 or 3.2 software. Real-Time qPCR data was collected and processed using Applied Biosystem 7500 Real-Time qPCR software. Image quantification was performed using FIJI (ImageJ). TopSpin 4.0 was used to process the NMR data. Chenomx 10.1 was used for metabolite quantification from ^1^H NMR data. Statistical analysis and graph plotting was performed using GraphPad Prism 9 for MacOS. Multivariate analysis such as principle component analysis (PCA), partial least squares discriminant analysis (PLS-DA), and variable importance plot (VIP) were performed using MetaboAnalyst 5.0 (https://www.metaboanalyst.ca/). Analysis of 16S sequencing data such as alpha- and beta-diversity as well as linear discriminant analysis effect size (LEfSe) was performed using MicrobiomeAnalyst (https://www.microbiomeanalyst.ca/). Figures were assembled in Affinity Designer (version 1.8.3, MacOS).

## Supplementary Material

Supplementary figures.

## Abbreviations

6-OHDA: 6-hydroxydopamine
ABX: antibiotic
B16F10: B16F10-Luc-GFP
BMRB: Biological Magnetic Resonance Bank
CCK-8: cell counting kit-8
dTMP: thymidine derivative
FMT: fecal microbial transplant
GSH: glutathione
HMDB: Human Metabolome Database
IACUC: Institutional Animal Care and Use Committee
IVIS: *in vivo* imaging system
LC/MS: liquid chromatography/mass spectrometry
LEfSe: linear discriminant analysis effect size
MM-LM: malignant melanoma lung metastasis
m/z: mass/charge
MTHFD2: methylenetetrahydrofolate dehydrogenase 2
NMR: nuclear magnetic resonance
NOESY: Nuclear Overhauser Effect Spectroscopy
PCA: principle component analysis
PDAC: pancreatic ductal adenocarcinoma
PDAC-LM: pancreatic ductal adenocarcinoma lung metastasis
PBS: phosphate-buffered saline
PLS-DA: partial least squares-discriminant analysis
SAM: S-adenosylmethionine
SCFA: simplest short-chain fatty acid
VIP: variable importance plot
