# Supplementary material for: Gut microbiota-derived formate exacerbates pulmonary metastasis in cancer
Source: Theranostics. 2025 Jul 2;15(15):7693–708. doi: 10.7150/thno.108873 (PMC12316040; doi:10.7150/thno.108873)
Supplement: Supplementary file 1 — Supplementary figures. [file thnov15p7693s1.pdf]

Supplementary Information

Gut microbiota-derived formate exacerbates pulmonary metastasis in cancer

Ray Putra Prajnamitra[1,*], Chaw Yee Beh[1,*], Wen-Hung Tang[1,*], Shu-Chian Ruan[1], Yuan-Yuan Cheng[1], Pei-Teng Kuo[1], Yu-Wen Tien[2], Patrick Ching-Ho Hsieh[1,3,#]

1. Institute of Biomedical Sciences, Academia Sinica, Taipei 115, Taiwan
2. Department of Surgery, National Taiwan University Hospital and National Taiwan University College of Medicine, Taipei 100, Taiwan
3. Institute of Medical Genomics and Proteomics and Institute of Clinical Medicine, National Taiwan University College of Medicine, Taipei 100, Taiwan

* Equal contributions.

[#] Corresponding author:

Patrick C.H. Hsieh, MD, PhD

Institute of Biomedical Sciences, Academia Sinica

128, Academia Road, Section 2, Nankang District, Taipei 115, Taiwan.

Phone: 886-2-27899170; Fax: 886-2-27853569

E-mail: phsieh@ibms.sinica.edu.tw

Supplementary Methods

Antibiotics Treatment

An antibiotics (ABX) cocktail containing ampicillin (Sigma, 250 mg/L), metronidazole (Sigma, 250 mg/L), neomycin (Sigma, 250 mg/L) and vancomycin (Sigma, 125 mg/L) was administered to mice via drinking water starting from when the mice reached 11 weeks of age for the ABX group or 10 weeks of age for the FMT group. For the ABX group, seven days after the ABX treatment commenced, lung metastasis was induced and the ABX treatment was continued until the experimental point. The ABX drinking solution was changed every two days to ensure freshness and cages were changed daily to ensure sterility.

Fecal Microbiome Transplantation (FMT) Procedure

Freshly collected stool pellets (150 mg) from non-ABX-treated, age- and gender-matched control mice were cut into small pieces and suspended in 3 mL freshly-sterilized PBS. The suspension was vigorously vortexed and spun down. The supernatant was collected and fecal solution (200 μL) was carefully administered to each mouse *via* oral gavage using polypropylene feeding tubes (Instech Lab). At the end of the 7-day ABX treatment, the mice were given normal drinking water for one day (assigned as day 0) and fecal transplant was performed on days 1, 3 and 5. On day 7, the mice were subjected to lung metastasis induction.

Formate Supplementation Procedure

Formate (Sigma) solutions for drinking (17 g/L) and injection (68 g/L) were prepared and sterilized. Formate treatment was started when the mice reached 11 weeks of age. It was administered by drinking water and daily intraperitoneal injection (500 μL). Seven days after the formate treatment commenced, lung metastasis was induced and the formate treatment was continued until the experimental point.

Validation of NMR Sensitivity and Specificity

To assess the sensitivity and spectral resolution of our NMR protocol, standard mixtures containing sodium formate, sodium acetate, sodium propionate, and sodium butyrate were prepared at three concentrations (10, 100, and 1000 μM) in phosphate buffer (pH 7.4) prepared in 10% D_2O with 40 μM TSP. Samples were analyzed on a Bruker NEO 500 MHz NMR spectrometer using the standard 1D NOESY pulse sequence with water suppression. Spectra were processed using TopSpin 4.0, and chemical shifts were confirmed using reference data. Peak intensities were normalized to the TSP peak, and spectral regions between 0–3 ppm and 8.3–8.6 ppm were inspected to evaluate resolution and overlap.

Formate Detection in Conditioned Media of Melanoma Cells

To assess whether melanoma cells produce formate, B16F10 cells were seeded in 6-well plate at different density in standard DMEM containing 10% FBS and cultured for 24 h. Conditioned media were collected. Amicon ultracentrifugation tubes (MWCO 3 kDa) were washed three times with sterile ddH_2O. Conditioned media were then filtered through the tubes by centrifugation (15000 rpm, 2 h, 4 °C) and the supernatant was collected. Into the samples were added phosphate buffer solution (100 mM) in 10% D_2O containing TSP (40 μM). Samples were then transferred to 5 mm NMR tubes and the NMR spectrum was recorded using a Bruker NEO 500 MHz ^{1}H-NMR spectroscopy. Spectra were processed using TopSpin 4.0 software, and the presence of formate was identified by a singlet peak at δ ~8.44 ppm.

Pancreatic Ductal Adenocarcinoma Lung Metastasis Model

<u>Culture and Inoculation</u>

Luciferase- and GFP-expressing KPC (B16F10-Luc-KPC) pancreatic cancer cells were cultured in Roswell Park Memorial Institute (RPMI) 1640 culture medium supplemented with 10% FBS, L-glutamine, sodium pyruvate and non-essential amino acids. On the day of inoculation, 5×10^5 cells

suspended in 100 μL sterile PBS were injected into the tail vein of 12-week-old C57BL/6 mice.

In Vivo Monitoring

Tumor growth was monitored weekly through IVIS (PerkinElmer) starting from day 7 post injection. Mice were anesthetized through isoflurane inhalation and intraperitoneally injected with D-luciferin firefly potassium salt solution (200 μL, 15 mg/mL in PBS, Biosynth). A series of IVIS images was then captured (exposure time 60 s, F/stop 1) until the bioluminescence intensity of each mouse reached the maxima. Body weight was monitored weekly starting on the day of cell injection throughout the experiment.

Ex Vivo Tumor Burden Measurement

On day 21, mice were sacrificed and lungs were excised and imaged by bioluminescence imaging to quantify tumor burden. The lungs were placed on a dish and D-luciferin firefly potassium salt solution (1 mL, 300 μg/mL in PBS, Biosynth) was added to cover the whole lungs. A series of IVIS images was then captured (exposure time 60 s, F/stop 4) until the bioluminescence intensity of each lung sample reached the maxima. The lungs were then preserved in 4% paraformaldehyde, sectioned (5 μm) and subjected to H&E staining for tumor burden quantification.

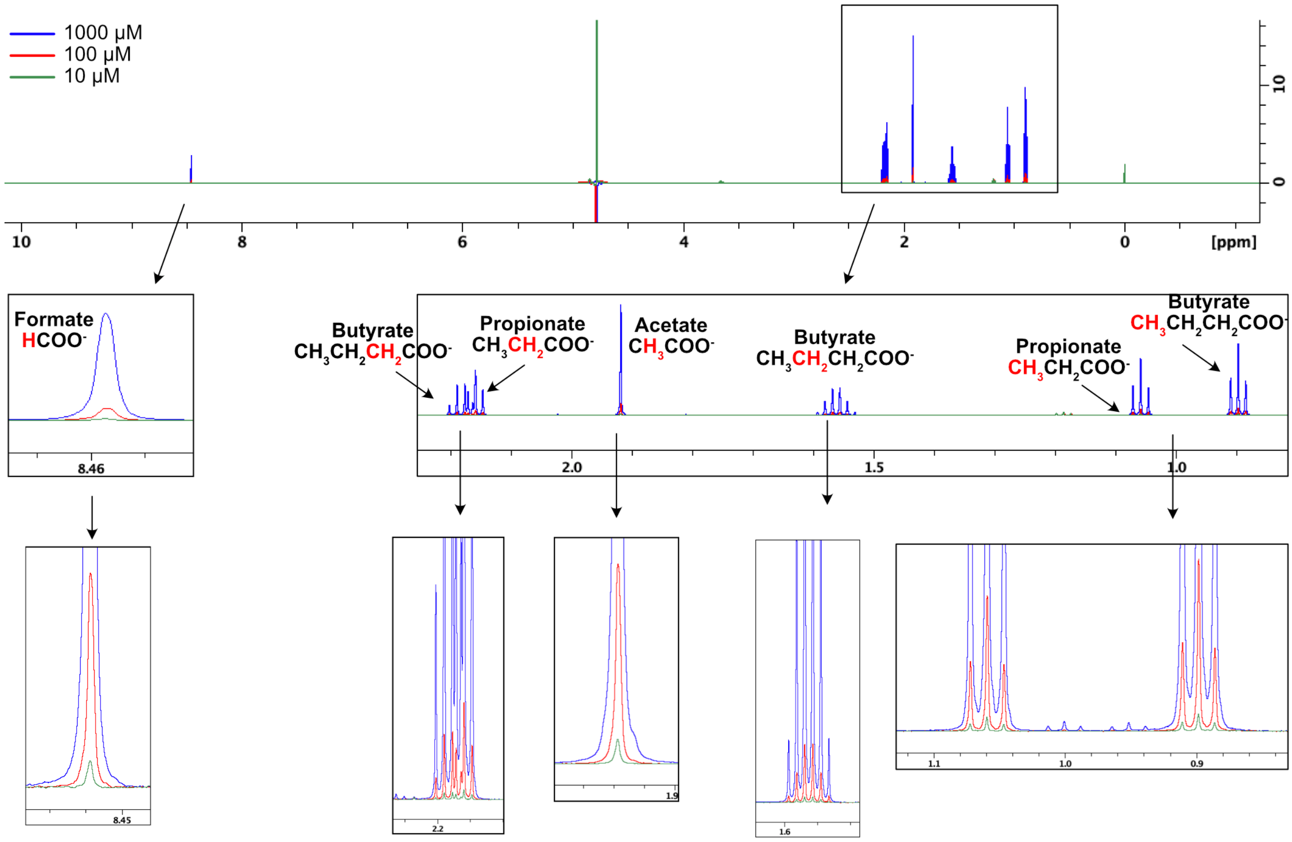

Supplementary Figure 1. Validation of NMR sensitivity and spectral specificity.

Overlay of normalized ^{1}H NMR spectra from SCFA mixtures at physiologically-relevant concentrations of 10, 100, and 1000 μM. Enlarged view of the ~8.44 ppm region highlights the formate peak, also detectable at 10 μM. Enlarged region from 0–3 ppm shows acetate, propionate, and butyrate peaks, all of which were also detectable at 10 μM. These results confirm that NMR spectroscopy reliably detects and distinguishes formate from structurally similar SCFAs at physiologically relevant concentrations.

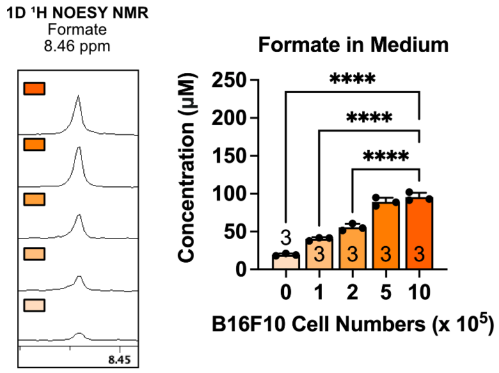

Supplementary Figure 2. Melanoma cells produce formate *in vitro*.

B16F10 melanoma cells were cultured in standard DMEM for 24 h. Conditioned media were collected and analyzed using 500 MHz ^{1}H-NMR spectroscopy with TSP as internal reference. A prominent formate peak at ~8.44 ppm was detected in the conditioned media but only in very low concentration in blank DMEM, indicating that B16F10 cells produce and release formate during proliferation. Analyzed by one-way analysis of variance with Tukey's correction. ****$P < 0.001$.

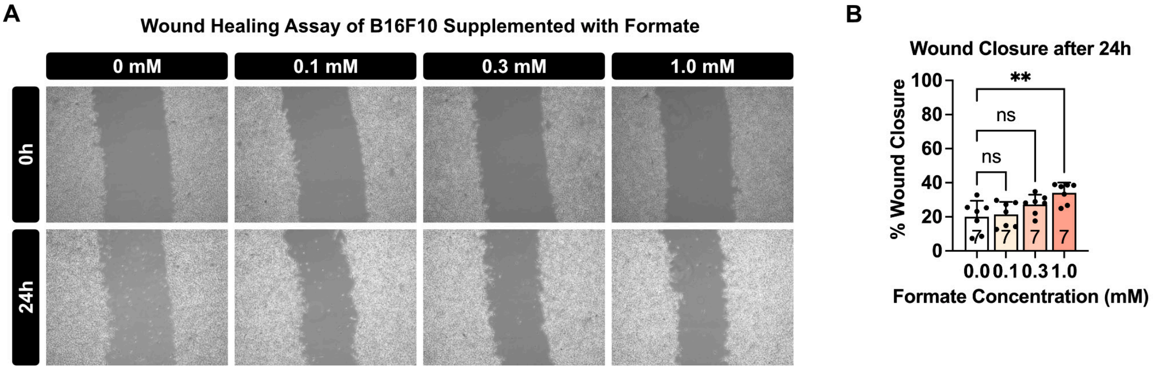

Supplementary Figure 3. Wound healing assay of B16F10 with formate supplementation. (**A**) Representative images of wound closure with different formate dose after 24 h incubation. (**B**) Quantification of wound closure, analyzed by one-way analysis of variance with Dunnett's correction. $^{**}P < 0.01$; ns, not significant.

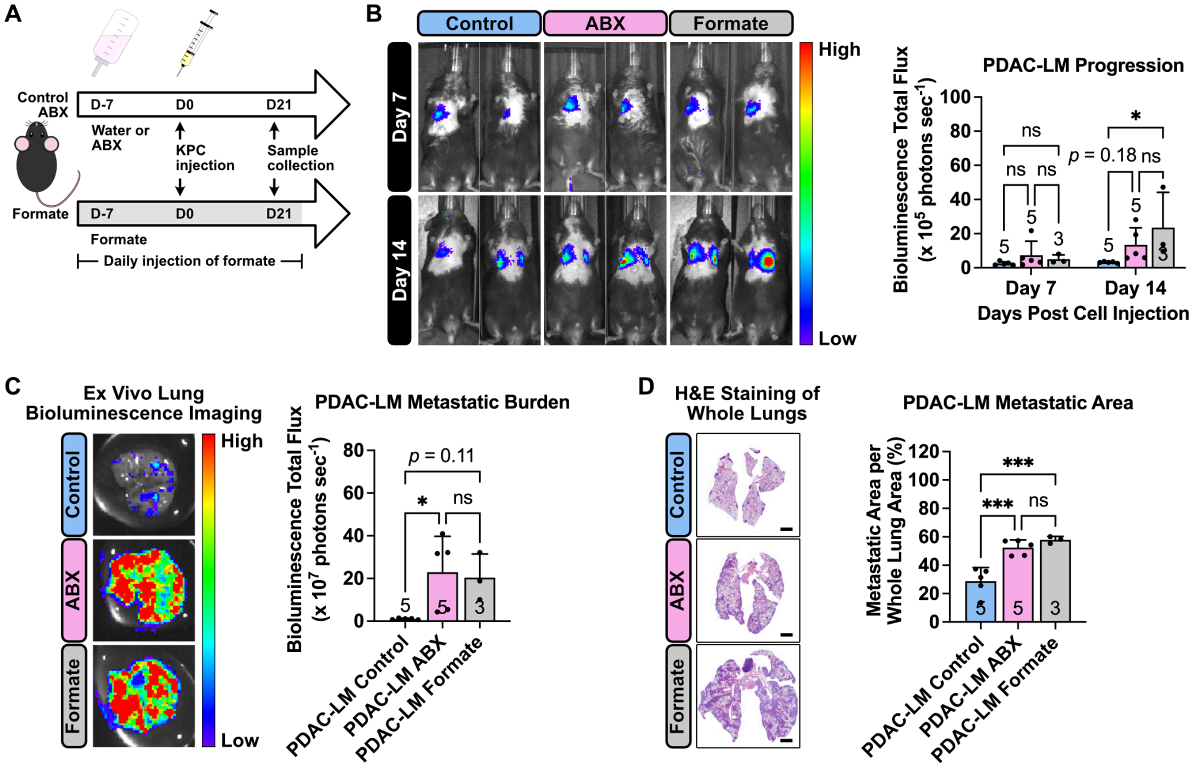

Supplementary Figure 4. Formate supplementation increases PDAC metastasis aggressiveness. (**A**) Mice were given formate supplementation to increase the amount of circulating formate prior to cell inoculation. On the day of tumor inoculation, 5×10^5 KPC cells were injected through the tail vein. Formate supplementation and ABX treatment were continued until the end of the experiment. (**B**) IVIS was used to monitor tumor progression, analyzed by two-way analysis of variance with Tukey's correction. (**C**) *Ex vivo* bioluminescence imaging was used to determine lung metastasis burden, analyzed by one-way analysis of variance with Tukey's correction. (**D**) H&E staining of excised lungs was used to determine the metastatic area in the lungs, analyzed by one-way analysis of variance with Tukey's correction. Scale bar: 2 mm. *$P < 0.05$; ***$P < 0.001$; ns, not significant.

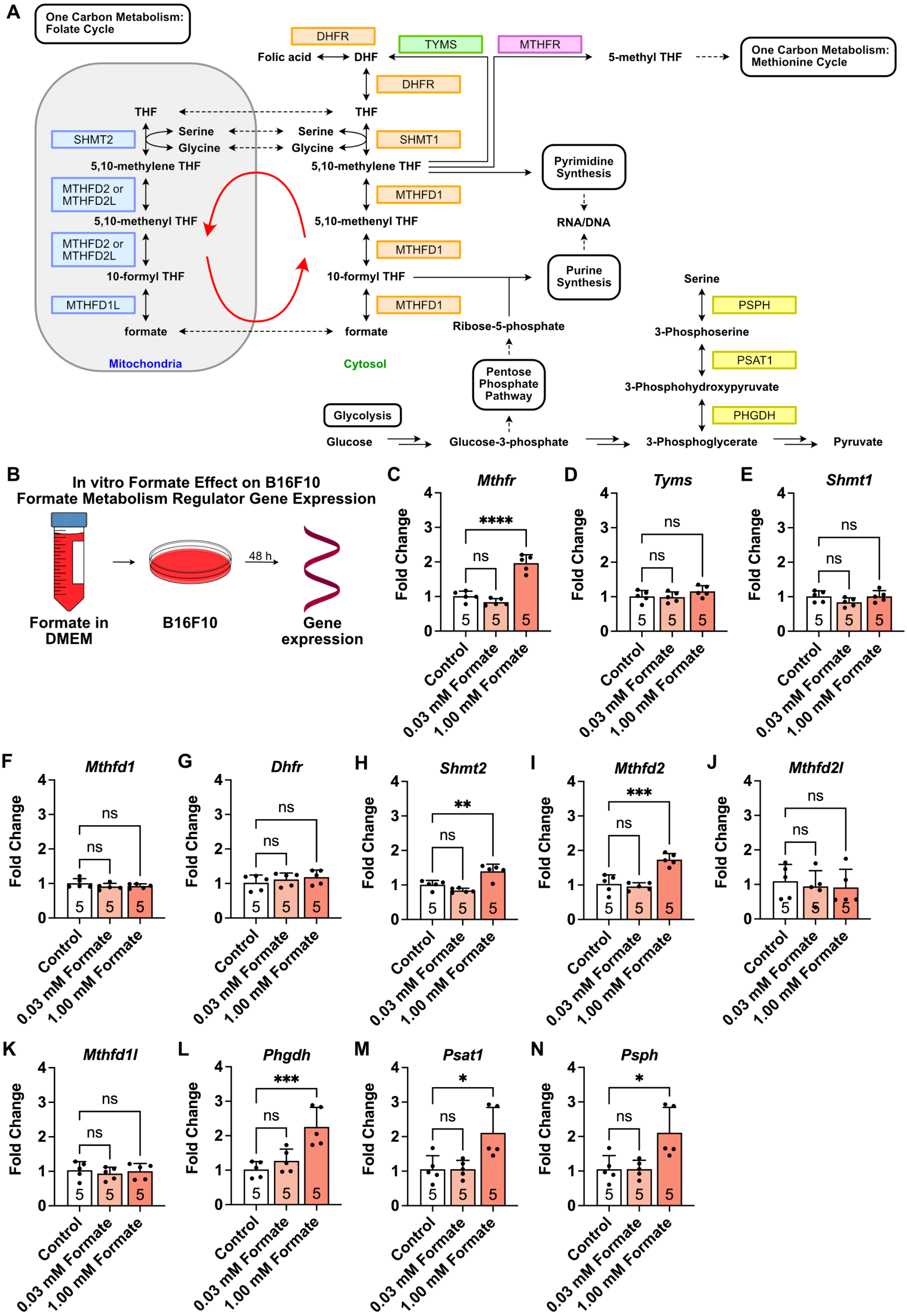

Supplementary Figure 5. One carbon metabolism-related gene expression changes in B16F10 melanoma cells post formate supplementation. (**A**) One carbon metabolism and related pathways. (**B**) Workflow of the experiment: B16F10 melanoma cells were incubated in formate-supplemented

DMEM for 48 h, followed by gene expression analysis. (**C-N**) Relative genes expression of one carbon metabolism and related pathways, analyzed by one-way analysis of variance with Dunnett's correction. $^{*}P < 0.05$; $^{**}P < 0.01$; $^{***}P < 0.001$; $^{****}P < 0.0001$; ns, not significant.
